# Supplementary material for: Switching state-space modeling of neural signal dynamics
Source: PLoS Comput Biol. 2023 Aug 28;19(8):e1011395. doi: 10.1371/journal.pcbi.1011395 (PMC10491408; doi:10.1371/journal.pcbi.1011395)
Supplement: Attachment — Submitted filename: response_letter.pdf [file pcbi.1011395.s005.pdf]

Response to the Reviewer Comments on PCOMPBIOL-D-22-01692 "Switching state-space modeling of neural signal dynamics"

Mingjian He, Proloy Das, Gladia Hotan, and Patrick L. Purdon

Preface

This is a revised version of our manuscript "Switching state-space modeling of neural signal dynamics" (PCOMPBIOL-D-22-01692). Based on the comments of reviewers, we have now substantially revised our manuscript by including new simulation analyses, elaborating on the EEG sleep spindle analysis and its interpretations, improving our flow of presentation and word choices, and contextualizing our work within other switching methods in the field. In summary, the major changes in this revision include:

1) Added simulation studies exploring
   a. sample size requirements (**Fig 3c**, **Fig S4-4c**),
   b. convergence of negative free energy (**Fig S4-1**, **Fig S4-2**) and model parameters to their stationary values (**Fig 3d**, **Fig S4-4d**),
   c. effect of less informative initialization, i.e., initialization using the same distributions with wider support for different switching models (**Fig S4-4** in comparison to **Fig 3**),
   d. comparison of our closed-form variational switching state-space inference against black-box variational switching state-space inference methods available online (**Fig S4-6**), and
   e. scalability of our method to increasing hidden state dimension and number of switching states in the context of neural oscillations (**Fig 6b**,**c**,**d**).
2) Restructured the subsection **Real application: spindle detection** into two subsections:
   a. **Switching state-space oscillator models**, where we present an additional simulation study using oscillator models to validate the model class used in the spindle detection (**Fig 6**);
   b. **Real-world application: spindle detection**, which is revised with interpretations of by-products of the time-domain analysis in addition to segmentation, i.e., model parameters and estimated hidden states (**Fig 7**). We now also contrast our segmentation with that of a popular wavelet-based spindle detection method that is often employed as a proxy for human expert hand-scoring (**Fig 8**). Finally, we alluded to possible soft and hard segmentation strategies (**Fig S4-7**) and real-time detection of spindles (**Fig S4-8**).
3) Added clarifications on a few detailed topics, including the derivation of interpolated densities, the behavior of fixed-point iterations to polarize model responsibilities to extreme values (i.e., 0 or 1), and the use of fixed observation matrices to make the presentation clearer to readers.
4) Carefully reworded several of our statements to explicitly state the relations of our work to Ghahramani and Hinton 2000, and replaced our improper uses of 'non-stationary', 'exact inference of posterior' by 'time-varying', 'efficient closed-form inference', respectively, to be more accurate.
5) Substantially improved the **Discussion** section with additional elaborations on: the similarities and distinctions of our method from other existing methods including black-box variational inference, suitability of one approach over the other in different contexts, and potential extensions to non-Gaussian observation models.

Finally, we are really grateful to the Associate Editor and the reviewers for constructive feedback to improve the quality and scope of our work. We believe that the manuscript has been strengthened considerably as a result. In what follows, we respond (in black) to each of the reviewers' comments (in green). The corresponding changes to the manuscript are highlighted in blue for ease of tracking.

Response to the Comments of Reviewer 1

Manuscript by He and coworkers thoroughly describes a statistical method for time series segmentation. Switching linear state space modeling framework that combines linear-gaussian observation, linear-gaussian processes, and hidden-markov-model is a simple yet useful model for seemingly non-stationary processes. Although they have been known for decades, only recently, there has been a wider acceptance of such methods in neuroscience. The manuscript is well-written and has high pedagogical value as it meticulously describes the model, derivation, and numerical procedures. In my opinion, the main contributions of the work are in the methods and appendix sections. The main contribution is the new initialization scheme and mathematical elegance of the model that makes the switching linear state space model usable. Although the methods are evaluated for neural time series segmentation, the inference framework is general and can be used for estimating the continuous latent states as well. The code is made available online with the BSD 3-Clause Clear License.

Response: We would like to thank Reviewer 1 for their careful evaluation of our work and valuable feedback.

Comments:

1. I enjoyed reading the manuscript very much.

Response: We are very grateful for the comment.

2. I have an issue with the word "non-stationary". "Time-varying activity" is not a synonym with "non-stationary" process. In the strict mathematical definition of stationarity, all models and their marginals described in the paper are stationary, as the joint probability law are shift invariant in time. Stationary models can have transient events as long as the statistics of the transient events are time invariant.

Response: We appreciate the reviewer for pointing this out. Indeed, we had meant to use "non-stationary" to refer to time-varying activity. All the models considered in this work are doubly-stochastic stationary time-series models. Many time-varying neural activity can indeed be well described by doubly-stochastic stationary processes. Acknowledging this distinction, we have now replaced all mentions of "non-stationary" by "time-varying".

3. A major weakness of the numerical results is the fixed observation matrix. The writing does not remark this fact. There are invariances that are taken care of with such assumption, and with sufficient domain knowledge it can be justified. However, this may not be the case in practice, and the inference framework presented does not assume a fixed observation matrix (eq. 28). I would like to see discussions regarding this.

Response: We understand the reviewer's concern and realize that we previously neglected this point in our discussion. As the reviewer pointed out, we have provided the M-step update rules for observation matrices, with Eq. 28 in the main text and Eq. 11 in **S2 Appendix. Joint estimation of parameters**. We now discuss in the **Discussion** section (line 466-480, p. 29) that we opted to use fixed observation matrices in our analyses to facilitate recovering a unique solution.

4. Another major weakness of the numerical results is the lack of convergence studies. As a function of dataset size, how quickly do the parameters converge? For example, Fig 3. b, the distribution of R for VI-I inference seems to be almost the same as the initialization. Why is this the case? Does making the dataset longer resolve this? How much data is needed to have clearly superior segmentation results?

Response: We appreciate the reviewer's suggestions to add more convergence studies. Accordingly, we have now performed simulation studies showing:

1. how percentage correct segmentation changes with varying data lengths (**Fig 3c**);

2. how model parameters converge to stationary points during EM iterations (**Fig 3d**).

We also repeated the same experiment in **S4 Appendix. Additional analyses** with wider support of initial value distributions, with convergence results shown in **Fig S4-4d**. All these results suggest that increasing the data length reduces model parameter estimation errors while requiring slightly more EM iterations for convergence. On the other hand, the model parameters converge quickly under 5 EM iterations to their stationary values, admitting smaller estimation errors for VI-I EM than VI-A EM.

Regarding the reviewer's specific questions on **Fig 3b**, the observation noise variance, R, does not vary much during EM iterations since its magnitude is much smaller compared to the state noise variances, $\mathbf{Q}$. Therefore, the negative variational free energy profile is considerably less sensitive to the changes in R, compared to the changes in Q (see section **Relative sensitivity of parameter updates** and **Fig S4-3** in **S4 Appendix. Additional analyses**). Increasing the data length does not resolve this relative insensitivity.

Lastly, the amount of data needed to have superior segmentation results will depend on how close the underlying switching models are. But, in all of our simulation studies, VI-EM is able to outperform the other inference algorithms even on the shortest data length we examined (**Fig 3c** and **Fig S4-4c**).

5. Fig 5. Only the joint VI-I is plotted. I would like to see the original parameterization as well.

Response: We left out the distribution plots of learned parameters for simplicity in this bivariate AR1 simulation study. Following the reviewer's suggestion, we have now included the distribution plots of learned parameters for this bivariate AR1 simulation study as **Fig S4-5** in **S4 Appendix. Additional analyses** and pointed readers to these results from the corresponding portion of the **Results** section.

6. The result section writing is not as smooth as the methods and appendices. Improvements in writing for presentation is suggested.

Response: We appreciate the careful read and feedback. We have now gone through the **Results** sections carefully and edited our exposition of the results to flow more smoothly across the subsections.

7. The EEG sleep spindle analysis is underwhelming. I would like to see the inferred parameters (with variational posterior distribution) and interpretations. Does the segmentation match expert evaluation? How predictive is the model?

Response: We would like to thank the reviewer for pointing out our restricted focus on only spindle segmentation and overlooking the interesting *by-products* of our time-domain approach. Besides estimating posterior model probabilities that we used for segmentation and spindle detection, our time-domain approach provides other parametric characterizations of spindle activity, like the center frequencies of the spindle activity. Additionally, the underlying spindle (and slow oscillation) waveform can be extracted via the inference procedure without any bandpass filtering. While the spindle waveform appears similar to the bandpass filtered signal, unlike bandpass filtering we can construct confidence intervals around the estimated spindle activity.

To highlight these important by-products, We have now updated in **Fig 7 and 8** with the estimated spindle time-series with 95% confidence intervals and added the inferred spindle center frequencies to the panels. Furthermore, the current method naturally limited spindle activity to the statistically relevant periods without arbitrary thresholding as shown in **Fig 7 and 8**. Lastly, we note that these estimated hidden oscillator states (i.e., the real and imaginary parts) can be readily used to compute instantaneous amplitude and phase for downstream hypothesis testing.

To compare with human expert hand-scoring, we used a traditional spindle detection algorithm based on wavelet transform (Wamsley et. al, 2012) as a proxy, since this algorithm has been shown to correspond well to expert scoring and frequently used in studies of sleep spindles. We now demarcate the time periods marked as spindle activity by this popular spindle detection method in **Fig 8**. The comparisons show that every spindle identified by the conventional method was also captured by VI-I EM. However, there

were many other spindle events labelled by VI-I EM that were missed by the current "clinical standard". These events nevertheless showed obvious sigma-frequency power, suggesting a potentially inadequate detection by expert scoring. Furthermore, wavelet-based detection relied on arbitrary thresholds that only selected the strongest portion of a spindle activity. In contrast, actual spindles likely lasted for a longer duration, as evident both from the spindle waveforms estimated by VI-I EM and from the original signal time traces. We have now incorporated these results in the **Real-world application: spindle detection** subsection (pp. 22-24) in **Results** and the corresponding **Discussion** section (p. 31).

Finally, we were unsure what the reviewer meant by "predictive"-ness of the model. We interpreted *predictiveness* as the ability of this switching state-space model to detect spindles in real time. Within our framework, it is possible to assign probability to every time point if the current observation comes from a spindle neural state, given all past observations. So after every observation is made, we have the ability to decide if spindle activity is present or not. However, this is not forecasting the spindle event by any means, since we are using all data up to and including the current observation. We have included an example of such *filtered* estimates of spindle probability as **Fig S4-8** in **S4 Appendix. Additional analyses** and pointed readers to this section from our **Discussion** section. Many false positives with very short duration are present, likely a result of window edge effects of variational inference on isolated abrupt changes in the time trace. However, the method is able to flag a spindle consistently with about 100 ms delay.

8. The model used in spindle analysis is different from that in the simulation studies. This weakens the paper.

Response: We understand that there is value in using the same model class in both simulation and real data analysis studies. However, we also wanted to demonstrate the efficacy of our method on a difficult problem, namely the AR1 model segmentation problem, that had been studied in the previous work by Ghahramani and Hinton 2000. Nonetheless, we understand the reviewer's concern, so we have added a new subsection titled **Switching state-space oscillator models** (pp. 18-20) under the **Results** section on a simulation study using oscillator models prior to the spindle detection study. In this added subsection, we only investigated the segmentation inference when the true parameters are known, since parameter learning has already been addressed in previous subsections, and oscillator models can be well-initialized in practice as we discuss in the subsection (line 281-286, p. 20).

9. Fig 6. why are VI-I and VI-A posterior states so sharp in transition between 0 and 1? It looks like only those were thresholded at 0.5 and the other methods are reporting probability of spindle state directly.

Response: We appreciate the reviewer's careful attention to this detail. We assure the reviewer that even though they appeared to be thresholded at 0.5, we did not apply any thresholding. The model probabilities behave this way because of a subtle feature of the variational approximation solution to the problem. The fixed-point iterations of the variational approximation polarize the model responsibility $\mathbf{h}_t$, making these posterior switching state probability estimates close to either 0 or 1. More specifically, a smaller (bigger) value of $\mathbf{h}_t^{(m)}$ allows less (more) contribution from $\mathbf{y}_t$ to the Kalman smoothing estimates corresponding to the mth candidate model, resulting in an even smaller (bigger) value of $\mathbf{h}_t^{(m)}$ at the next iteration. This is because the Kalman smoothing weighs the observation noise $\mathbf{R}$ by $1/\mathbf{h}_t$, thus a larger $\mathbf{h}_t$ relative to the other candidate model(s) gets pushed to 1 while others get pushed to 0. Right at the transition boundaries, some of the posterior model probabilities can be around 0.5 but they are not changing gradually as in the other filtering methods. We have now added this explanation to the **Materials and methods** section (p. 43), as it adds to a better understanding of the switching inference solution. We would like to thank the reviewer for this helpful comment.

Additionally, once the interpolated densities are obtained with VI-I EM, we can consider the interpolated densities as (being proportional to) the emission probabilities to perform a soft segmentation based on the forward-backward algorithm, and a hard segmentation based on the Viterbi algorithm. We have included these results as **Figure S4-7** in **S4 Appendix. Additional analyses** for completeness.

10. p22. I disagree that you showed "extensive simulation results". The first two simulations were model matched validations. Only the third simulation provides some extra value. If the sample size analysis is added, perhaps it could be justified.

Response: Following this suggestion and previous comments, we have now included additional simulation studies in the main text showing:

1. how percentage correct segmentation changes with varying data lengths (**Fig 3c**);
2. how model parameters converge to stationary points during EM iterations (**Fig 3d**).

We have also added a new subsection titled **Switching state-space oscillator models** (pp. 18-20) on a simulation study using oscillator models where we investigated how segmentation performance scales with increasing number of possible switching states (**Fig 6**).

In addition, we added a new appendix (**S4 Appendix. Additional analyses**) with additional analyses on the existing simulation studies. In this appendix, we:

1. showed negative free energy trajectories during EM iterations (**Fig S4-1 and S4-2**),
2. repeated the same simulation study that generated **Fig 3** with less informative parameter initialization (**Fig S4-4**), and
3. showed the distributions of estimated model parameters for the simulation study in **Fig 5**.

11. Discussion compares to relevant other methods. One thing that should be mentioned is the handling of the non-gaussian likelihoods. Many methods that are designed for neural spike train observations can handle Poisson process or other observation models.

Response: We thank the reviewer for the comment. We now mention how the proposed method can be extended to non-Gaussian observation models in the **Discussion** section (pp. 29-30). Specifically, we point out that the proposed method can handle observations generated by counting processes (e.g., point processes to model neural spiking activity with history dependence) by replacing the Kalman filter with extended Kalman filter (Smith and Brown, 2003) or unscented Kalman filter (Wan et. al., 2000).

12. p 36. "HMM with `exp{g_t^(m)}` as observation probabilitie" `<==` normalization is necessary for such interpretation.

Response: We are grateful to the reviewer for their careful reading. The phrase is replaced by "an HMM with observation probabilities proportional to $\exp\{g_t^{(m)}\}$" (p. 41).

13. p 41. M-step is closed-form, why say "convex optimization"?

Response: Even though we derived closed-form M-step update equations, the M-step equations are maximizing the negative variational free energy computed with respect to the current posterior distribution estimates (shown in Eq. 14) over the distributional parameter space. In other words, M-steps are in fact part of the iterations that recursively maximizes the expression in Eq. 14. We mentioned 'convex optimization' in that context and prefer to keep the wording to be general and to allude to the possibility of using stochastic gradient descent or other optimization techniques when the M-step update equations cannot be derived in closed-form, such as under non-linear or certain non-Gaussian scenarios.

14. S3 p4. It wasn't immediately clear to me that the interpolated densities corresponded to the equation. Perhaps adding an intermediate step could help.

Response: We now add the definition of interpolated densities and the intermediate steps that follow from Theorem 4 of de Jong 1989 (p. 4 of **S3 Appendix. Implementation details**).

15. The variational inference for each of the parameterized joint model seems to require some math. Please add a comment on how this may or may not be easily automated (perhaps using autodiff and black-box variational techniques)

Response: As the reviewer pointed out, switching inference and variational learning for each of the parameterized joint models requires some math. Concretely, the computations have two separate layers:

- the switching inference layer that uses well-known message passing algorithms with little modifications, and
- the parameter update layer that requires some explicit calculations involving matrix differentiation.

Following the reviewer's comment, we note that the gradient can indeed be obtained by using autodiff, and consequently used to update the model parameters using gradient descent. However, that automation will result in an iterative M-step, losing the benefits of having fast closed-form updates for every parameter within a single iteration.

Regarding the black-box variational techniques, they are certainly possible in the context of switching state-space models (see Linderman et. al, 2017). However, to fully exploit our existing domain knowledge through constructing specific shared dynamics as we have done in the sleep spindle detection analyses, one needs to explicitly define the structures. And once the structure is defined, derivation of the corresponding M-step equations is not difficult. In the worst case scenario, one can perform gradient descent using automatically computed gradients, however that will increase the computational costs as explained above. We have added these points to the **Discussion** section (pp. 29-30).

Having said that, we have also performed a simulation study comparing several of the black-box variational techniques (mean field approximation, tridiagonal Hessian approximation, and Laplace approximation with structured posterior) to VI-I EM algorithm. The results show superior segmentation accuracy of VI-I EM compared to those black-box variational inference algorithms (see **Fig S4-6** in **S4 Appendix. Additional analyses**).

Response to the Comments of Reviewer 2

In this paper, the authors advocate using switching state space models for modeling neural dynamics. They describe the specific switching state model under study and a variational inference algorithm used to fit the model. Methodologically, they then contribute a novel initialization method for the variational inference algorithm. The initialization procedure is validated and explored in simulated experiments, where they show improved recovery of ground truth segmentation and parameter learning compared to the previous initialization procedure. The authors then apply their model to sleep spindle detection.

The high-level approach of considering efficient and accurate inference algorithms for switching state space models of neural data is a worthwhile pursuit. Additionally, the proposed initialization procedure is interesting and promising. However, the paper could be improved in a number of areas described in detail below. Namely, the clarity of the contribution, situation of the work in the literature, and simulated experiments could be improved with some modifications.

Response: We would like to thank Reviewer 2 for the careful evaluation of our work and for providing constructive suggestions to improve it.

Major comments and questions

1. It appears that the model and variational inference method are the same as those presented in Ghahramani and Hinton, 2000. However, at first I was under the impression that the proposed inference algorithm was developed by the authors in this paper. I suggest that the authors attempt to edit portions of the manuscript to make the relationship between the algorithm derived in this paper and the algorithm in Ghahramani and Hinton, 2000 more immediately clear. First, either 1) a statement saying the algorithm is the same as Ghahramani and Hinton or 2) these are the differences between the two algorithms (outside of initialization) would be helpful. Additionally, if it is correct that it is the same algorithm, the wording introducing the algorithm should be adjusted because it currently reads as if the authors developed this algorithm.
    (a) For example, the abstract line "Here we derive a solution to the inference problem.." could be removed, and a new line describing the contribution as a new initialization procedure for a variational inference algorithm from [Ghahramani and Hinton, 2020] placed instead.

Response: We understand the reviewer's concern and have made several changes in the manuscript to clarify the relationship of our work to that of Ghahramani and Hinton, 2000:

1. We have revised the **Abstract** (p. 1) to read "Here we revisit a switching state-space model inference approach first proposed by Ghahramani and Hinton", further adding that "we provide explicit derivations for solving the inference problem iteratively after applying variational approximation on the joint posterior of the hidden states and the switching process."

2. We also highlighted our new initialization procedure in the **Abstract** (p. 1): "We introduce a novel initialization procedure using an efficient leave-one-out strategy to compare among candidate models, which significantly improves performance compared to the existing method that relies on deterministic annealing."

3. In the last paragraph of the **Introduction** section (p. 5), we now explicitly say "we present a simple but rigorous derivation of the inference algorithm [42]" to be clear that the overall form of the algorithm is the same as in Ghahramani and Hinton 2000.

4. In the **Parameter learning using generalized EM algorithm** subsection when we refer to the flowchart of the algorithm (p. 43), we now explicitly mention that "It should be noted that while derivations differ, the overall form of this algorithm is the same as in [42]".

In the second and third paragraphs of the **Discussion** section on pp. 26-27, we discuss the similarities and distinctions of our derivation and initialization procedure from Ghahramani and Hinton 2000.

2. In simulation 2, the prior distributions used to sample initial parameter values could be improved. Currently, they are quite informative about the true parameter in that they are centered at the true parameter and typically come from non-overlapping distributions for the same parameter across states (e.g. F1 in U[0.8,1.0] and F2 in U[0.6,0.8]). I suspect this choice of distributions provides a lot of information that is unrealistic when the parameters are truly unknown. I suggest using distributions with wider support, not necessarily centered at the true value, and that the same parameter across states be drawn from the same distribution. These changes should help better assess how well each algorithm performs when the parameters are unknown.

Response: We took the reviewer's concerns into consideration and repeated the simulation study for less informative initial value distributions (see **Fig S4-4** in **S4 Appendix. Additional analyses**. Specifically, we drew the initial parameters of competing models from the same initial distributions with wider support. Despite of the slightly degraded segmentation accuracy, the results of this study replicate the patterns obtained in the original study in **Fig 3**. VI-I EM again outperformed the other methods (**Fig S4-4a**) with varying data lengths (**Fig S4-4c**). Consistent with the wider support of initial value distributions, the converged parameter estimates in **Fig S4-4b** have wider spread than **Fig 3b**. VI-I EM again achieved better parameter recovery than VI-A EM by skewing more towards the true values. The differences we noticed are that the parameter updates took more EM iterations to converge and on average incurred larger estimation errors compared to when informative distributions were used (**Fig S4-4d**). VI-I EM showed robust convergence of parameter estimates towards the true values, which improved further with a longer data length similar to the original simulations. We point readers to this simulation analysis from the corresponding portion of the **Results** section.

3. More importantly, I think the modifications described above for simulation two will also be important in evaluating the initialization procedure. As opposed to deterministic annealing, it seems as if the initialization procedure relies more strongly on having good estimation of the parameters early in inference, since it is based on log probabilities under the current LDS models. So while the proposed initialization procedure has shown promising performance in simulation 1 with the true parameters known, and in simulation 2 where the initial parameter values are quite informative about the true parameters, it will be important to see how it performs in a more difficult setting.

Response: We agree with the reviewer and find this comment very helpful. As we now show in pp. 4-5 of **S4 Appendix. Additional analyses**, using initial value distributions with wider supports (i.e., under a more difficult setting), VI-I EM is still able to provide superior segmentation accuracy over VI-A EM (see **Fig S4-4**) just as in **Fig 3**. Another interesting observation from **Fig 3** and **Fig S4-4** is that in VI-I EM, the segmentation accuracy improves with increasing data length while that is not the case for VI-A EM. This suggests that deterministic annealing is perhaps more vulnerable to the lack of prior information. This may be due to its tendency to get trapped in local maxima of the likelihood landscape.

In general, the variational inference algorithm described here is best suited for scenarios where an informed guess of model parameters can be used for initialization. From a neural signal processing perspective, we are equally concerned about characterizing interpretable hidden dynamics of different neural states and achieving accurate segmentation of time-varying activity due to transitions among them. This intention is reflected in our use of multiple models in parallel, which compete with each other to explain observed data, rather than using a larger array of models with arbitrary structures and parameterizations that are intended to approximate neural dynamics of interest. In contrast, the parallel models we use favor a more parsimonious and informed set of candidate models to obtain interpretable characterization of dynamics and accurate segmentation. The use of the interpolated density is consistent with this premise, since it better exploits prior domain knowledge and the carefully constructed candidate models. In cases where there is an unknown number of switching states, the dynamics are completely unknown, or the goal is to fit to data as well as possible, a number of black-box algorithms such as in Fox et al. 2011, Linderman et al. 2017, Zoltowski et al., 2020, and Glaser et al. 2020 could be highly effective.

We have now incorporated this point into the **Discussion** section (pp. 29-30) to better contextualize our work in the burgeoning field of switching state-space models.

4. In the section "extensions of model structure and parameter estimation" (line 184), it is worth noting that the authors are describing a switching linear dynamical system as described e.g. in ref 64, where the inference algorithm is designed to handle switches in the dynamics parameters. Therefore It would be helpful to compare an algorithm such as the one in ref 64. Additionally, I suggest changing initial parameter value distributions in this example as in the previous simulation.

Response: We have now included in **S4 Appendix. Additional analyses** an analysis comparing the VI-I EM segmentation accuracy with three switching state-space inference methods available on https://github.com/lindermanlab/ssm (Glaser et al. 2020). We used stochastic variational inference with mean-field approximation, posteriors with tridiagonal Hessian approximation, and Laplace-EM approximation on structured posterior. We note that in the last approximation, the structured posterior has the same functional form as our approximate distribution q.

The results are shown in **Fig S4-6**: VI-I EM is able to achieve superior segmentation accuracy compared to these algorithms. However, it should be noted that the three variational algorithms tested here used AR-HMM to initialize the model parameters from the data, and this initialization performed better than specifying the model parameters at the true values (results are not shown). On the other hand, VI-I EM started with initial model parameters that were informative of the true values, but had the advantage of exploiting specified model structure. In addition, the use of exact message passing and parameter updates along with interpolated densities in VI-I EM likely better exploits prior knowledge of the switching process. In essence, VI-I EM requires more informative candidate model construction compared to these other variational algorithms with more flexible approximations, resulting in better segmentation performance. We also included this discussion in **S4 Appendix. Additional analyses** on the differences of these three switching state-space inference methods from VI-I EM.

We would like to point out that in this case, both the candidate models share the same exact parameters, except for $\mathbf{F}_{12}$: in one candidate model it is 0; in the other model it assumes a non-zero value. Therefore, $\mathbf{F}_{12}$ is initialized for only one candidate model. The use of different initial value distributions in this simulation would not add more ambiguity between the two candidate models as it does in the previous simulations. The EM algorithm will just require more iterations to converge to the stationary values. Thus, we chose to stick with the current distributions given that we have explored the effect of less informative initialization in the previous simulation example, and that the results of **Fig S4-4** in **S4 Appendix. Additional analyses** resembled the original results of **Fig 3**.

Following this, we now articulate in the **Discussion** section (p. 30) about why the algorithm in this paper is best suited for applications where informed candidate models can be constructed based on domain knowledge and when the key objective is to obtain accurate segmentation and to refine characterization of distinct dynamics that can already be reasonably initialized.

5. The simulated examples and application all use models that are relatively low dimensionality in the state space. For example, it is often that there are 2 different LDS models each with 1 latent dimension. I would suggest that the authors explore how their initialization procedure performs as

the number of LDS models and number of dimensions grows, compared to deterministic annealing, to help determine the overall significance of the initialization contribution.

Response: We agree with the reviewer on the importance of exploring the scalability of the algorithm as the number of switching states and SSM dimensionality grow. We now include a new subsection titled **Switching state-space oscillator models** (pp. 18-20) where we considered simulations with up to 31 possible candidate models and with some candidate models having states up to 10 dimensions. These results are presented in **Fig 6** on p. 19, and they show that with increasing number of switching states, the accuracy of VI-A dropped precipitously while VI-I maintained good segmentation accuracy. This result suggests that our switching inference method is capable of modeling transient oscillations and is easily scalable with structured candidate models. VI-I was able to segment complicated switching trajectories close to the ground truth, such as in the example displayed in **Fig 6c** with 31 possible switching states from 5 oscillations, while VI-A failed to do so as shown in **Fig 6d**.

Minor comments

- Could cite additional uses of switching state space models for neural data: Petreska et al., 2011; Zoltowski et al., 2020

Response: These references are now incorporated in the manuscript.

- On pg. 33, I would recommend rephrasing "making exact inference possible". It could mislead a reader into thinking the overall inference method is exact, whereas this really refers to algorithm subcomponents.

Response: We appreciate the careful read. We have now revised the phrase to "On this approximate distribution, efficient closed-form inference can be performed" (see p. 38).
