# Supplementary material for: Switching state-space modeling of neural signal dynamics
Source: PLoS Comput Biol. 2023 Aug 28;19(8):e1011395. doi: 10.1371/journal.pcbi.1011395 (PMC10491408; doi:10.1371/journal.pcbi.1011395)
Supplement: Attachment — Submitted filename: response_letter.pdf [file pcbi.1011395.s006.pdf]

Response to the Reviewer Comments on PCOMPBIOL-D-22-01692R1 "Switching state-space modeling of neural signal dynamics"

Mingjian He, Proloy Das, Gladia Hotan, and Patrick L. Purdon

Preface

This is a re-submission of our significantly-revised version of manuscript "Switching state-space modeling of neural signal dynamics" (PCOMPBIOL-D-22-01692R1). Based on the comments of reviewers, we have made substantial revision of our manuscript. The major changes include:

1) We have now provided due credits to state-of-the-art Bayesian methods (Gibbs sampling based) in the switching state-space modeling literature and better contextualized our work within it in both **Introduction** and **Discussion** sections.

2) The flow of **Results** sections is restructured by bringing our novel initialization to the forefront and motivating our extensive simulation studies in that context to highlight it as a key contribution of the paper.

3) A detailed discussion on the polarized values of the model probabilities is now added to the **Materials and methods** section to help readers better understand the fixed-point iterations.

Finally, we are really grateful to the Associate Editor and the reviewers for constructive feedback to improve the quality and scope of our work. We believe that the manuscript has been strengthened considerably as a result. In what follows, we respond (in black) to each of the reviewers' comments (in green). The corresponding changes to the manuscript are highlighted in blue for ease of tracking.

Response to the Comments of Reviewer 1

Thank you for the updated manuscript. It addresses most of my concerns adequately. The additional analyses are much appreciated. I have just one remaining concern in response to the previous point no 9.

Response: We once again thank Reviewer 1 for their valuable feedback.

1. Posterior discrete state concentration during the fixed-point iteration

   It is intriguing that the approximate, factorized posterior tends to converge to a very sparse state through the fixed-point iteration inner loop. The form of the reciprocals is akin to the sparse Bayesian Gaussian regression and factor analysis procedures know as ARD. Automatic Relevance Determination (ARD) which is an iterative type-II maximum likelihood procedure leads to sparsification of coefficient in a similar iterative manner. If there's a relationship that can be shown, I suggest adding some discussion. ARD has been popularized by Bishop's PRML book and has been used in neuroscience widely.

Response: We appreciate the reviewer for pointing out the relevance of ARD to the fixed-point iterations. ARD is a procedure to determine which input features are most relevant in the classification/regression problem using the correlation length scales in a covariance function. The sparsification of the classification/regression coefficient in an iterative type-II maximum likelihood estimation follows from

the particular expression of the likelihood function. The mathematical construction of our problem is fundamentally different. Concretely, the fixed-point iterations in our problem updates the $h_t^{(m)}$ that is a deterministic summary statistics unlike the ARD procedure where the weight coefficients are randomly distributed with zero mean and a hyperpararmeter. The negative variational free energy expression (see S1 Appendix) involves $h_t^{(m)}$ differently from that of the ARD procedure (compare to [Bishop, 2006, Eq. 7.97]). In our problem, the empirical sparsification of $h_t^{(m)}$ is driven by the term $\mathbf{R}/h_t^{(m)}$ used in estimating the hidden model log-evidence, which is then used to compute $h_t^{(m)}$ at the next iteration: this procedure in turn pushes $h_t^{(m)}$ to either 1 or 0. Nevertheless, we find the analogy to ARD helpful for an intuitive understanding of the empirical effect of the fixed-point iterations, and we have thus included a reference to ARD in the **Materials and methods** section (lines 756-760, p. 43) when discussing this behavior of the fixed-point iterations.

2. Although it is nice to have a sparse selection of discrete states after the inference, this is clearly not always going to be close to the true posterior. I don't think this is necessarily a bad thing, but it needs to be made clear that the space of potential approximate posteriors are smaller than postulated by the explicit assumptions.

Response: The reviewer raised an interesting and subtle point that was not previously explicated in the text. The sparse model selection that follows our inference step may in fact be close to the true posterior, since the true posterior p is expected to contain polarized values (i.e., with model probabilities close to 1 or 0) under the assumed generative structure with parallel candidate models. This polarization is due to the following factors:

- First, each transition between candidate models introduces a *discontinuity* in the observation sequence at the switch point, as the realization trajectories from different SSMs are distinct from each other.
- Second, since the SSM hidden state trajectories consist of multiple time points, they reside in a high dimensional space. The dimensionality further increases as SSM state dimension increases. Because of this high-dimensionality, the separation between any two candidate models becomes very substantial.
- Third, real recordings often have infrequent transitions between distinct dynamics. This empirical skewness leads to a high probability to stay within the same candidate model. Such probability propagates as a strong prior across time points amplifying the effect of the other factors in polarizing the values of posterior estimates.

We did not mean to indicate that the reciprocal interdependence between $g_t^{(m)}$ and $h_t^{(m)}$ in the fixed-point iterations was designed to polarize $h_t^{(m)}$ estimates. Instead, it is derived by solving the optimization problem in Eq. 13, and it *could* recover sparse solutions when the true posterior is sparse.

Correspondingly, the space of potential approximate posteriors is indeed $\mathcal{P}$ as defined in Eq. 12 that includes non-sparse distributions, and is not a smaller subspace of $\mathcal{P}$. In fact, in both VI-EM and VA-EM algorithms, the values of $h_t^{(m)}$ are initialized away from 0 or 1 for the fixed-point iterations. Throughout the iterations, the values progressively become more polarized in both algorithms, in order to increase the negative variational free energy until reaching a local maximum. Thus, non-sparse models in the neighborhood of such local maximum are already considered in the search space during the fixed-point iterations. We have now edited the **Materials and methods** section (lines 737-748, p. 43) to include this explanation, and we appreciate the reviewer for helping us to clearly describe this key empirical behavior of the variational inference approach presented here.

3. The new methods hidden in S4, "Soft and hard segmentation with VI-I EM", can be useful but they do not represent principled derivations. How would one quantify the usefulness of the soft segmentation method? I would like to see some discussion for the practitioner. Also, please make sure the code includes these soft/hard segmentation options for ease of use.

Response: We thank the reviewer for the careful read. We acknowledge that both the soft and hard segmentation methods are ad hoc heuristics, in fact, they can be viewed as generalizations of the static

method derived in Bar-Shalom and Li [1993]. The static method uses the predicted density $p(y_t|x^{(m)}_{t|t-1})$ as a surrogate for the predicted observation probability $p(y_t|s_t = m, y_1, \cdots, y_{t-1})$ in an HMM. This is an approximation because the actual density $p(y_t|s_t = m, y_1, \cdots, y_{t-1})$ requires marginalizing out the entire history for $\{s_{t-1}, \cdots, s_1\}$ when computing $p(y_t|x^{(m)}_{t|t-1})$. In the same vein of approximation and Gaussian merging, we are approximating the smoothed density $p(y_t|s_t = m, y_1, \cdots, y_{t-1}, y_{t+1}, \cdots, y_T)$ with the interpolated density from each candidate model and running the forward-backward algorithm to obtain smoothed estimates of the switching state. Thus when viewed from this perspective, these soft/hard segmentation work in the same principle as the early switching state-space inference method.

The usefulness of the soft segmentation could be assessed empirically within a specific application, where one expects a gradual change in neural states as opposed to a sharp transition. For instance, a switch between two neural states may be driven by a covariate, perhaps a pharmacological agent that can be measured at a fine temporal resolution. Then relating variations of the covariate to the gradually changing model probability could be more practical. After all, the soft segmentation method emphasizes the transition periods between states acknowledging uncertainty in choosing one of the several underlying states rather than dividing a time series into multiple distinct segments with different dynamics. Thus, its usage is more relevant in applications where key hypotheses are focused on the transition periods between segments, than the segments themselves. We have added this discussion to the corresponding section in **S4 Appendix. Additional analyses** (last paragraph, p. 8).

We apologize for the delay in uploading the codes. Please see our somata github repo for the most recent changes. Both the soft and hard segmentation results are now outputted by default (see line # 163 of somata/switching/vb.py).

Minor:

- There are errors in the citation, e.g.,

  Glaser JI, Whiteway M, Cunningham JP, Paninski L, Linderman SW. Recurrent Switching Dynamical Systems Models for Multiple Interacting Neural Populations. Neuroscience; 2020.

  -->published in "Advances in Neural Information Processing Systems"

Response: Thanks for catching the typo. The reference [44] in p. 56 is now fixed in the manuscript.

Response to the Comments of Reviewer 2

Thank you for your thorough response and revisions. I still have some lingering, major concerns though.

Response: We once again thank Reviewer 2 for their constructive suggestions.

# Concern 1: Bringing interpolated density and parameter initialization to the fore

a. The revised manuscript makes clear that the methodological novelty is in the initialization scheme, but the main text gives very few details of the interpolated density initialization scheme. Instead, the details are punted to Supplement S3. Given that this is a core contribution, it should be a major component of the main text. The relative fraction of main text devoted to describing generalized EM as opposed to describing initialization strategies is not commensurate with the relative novelty of these two facets of the work.

Response: We understand the concern from the reviewer regarding emphasizing the novel initialization procedure in the manuscript. We have indeed aimed to highlight the interpolated density initialization as a major component of the main text, but it can be improved.

- First, a section is already devoted to the interpolated density in the **Materials and methods** section (lines 827-849, pp. 48-49), emphasizing the motivation and interpretation of this initialization. **S3 Appendix. Implementation details** covers the detail of actual computations of the interpolated density, which requires an exposition of Kalman smoothing recursions and some proofs we provide. We feel that explaining this implementation detail in the **Materials and methods** section instead may digress from understanding the use of interpolated density in our context.

- Second, we admit that the relative fraction of **Materials and methods** section that describes interpolated density is little when compared to the description of Generalized EM algorithm. However, we feel the complete derivation and explanation of this particular generalized EM framework for switching state-space models is an equally important contribution of this paper. We note throughout the manuscript that this idea was initially proposed by Ghahramani and Hinton [2000], but to the best of our knowledge no published work has provided straightforward derivations using optimal functional forms of variational log-posteriors to arrive at the fixed-point iterations. As we allude to in the **Introduction** section (lines 87-90, p. 5), all closed-form equations are presented here to help readers from an applied field (e.g., neuroscience) to understand switching state-space models, appreciate the statistical principles behind, and be able to implement and expand on the solutions we present. While this perhaps makes the **Materials and methods** section obvious and less interesting to an expert in statistics, we hope to keep the organization as is for its pedagogical values (which is noted and appreciated by Reviewer 1). Related to this, one way to highlight the importance of the initialization procedure in this framework is through explaining how the fixed-point iterations arise from optimizing the negative free energy and noting the challenges of a non-concave landscape. We now expand on this to motivate the importance of the initialization to further increase it weight (lines 761-768, pp. 43-44).
- Third, all **Results** sections are devoted to comparing switching inference and learning performance with and without the interpolated density during initialization. This focus demonstrates the improvements due to the novel initialization procedure and makes it a major component of the main text. We have added an explanation of the interpolate density in the leading paragraphs of the **Results** section (lines 129-141, p. 7) so that this novel initialization is better contextualized when the results are presented, perhaps before the **Materials and methods** section is perused. This change should further highlight the novel initialization as a core contribution of this manuscript.

b. A skeptical reader could ask whether the generalized EM algorithm is necessary at all, with reasonable initial estimates of the parameters. Given the same initial parameters $\theta^{(0)}$, one could used blocked Gibbs sampling (and many previous works have used blocked Gibbs to great success on SLDS, as the authors note). The analog of interpolated density initialization would seem be: initialize the continuous states with samples from the conditional distribution $p(x_{1:T}^{(m)}|y_{1:T}, s_t = m\ \forall t, \theta^{(0)})$, and then sample $s_{1:T}$ from its conditional distribution. With weak, conjugate priors on the parameters and good initialization, I would expect blocked Gibbs sampling to work very well. I realize that doing this comparison is non-trivial so I don't consider it a must-have, but I would personally be interested to know the outcome. (FWIW, https://github.com/mattjj/pyslds has Gibbs sampling with conjugate priors implemented for SLDS, so some of the annoying parts of working with priors on matrix-valued parameters could be ameliorated.)

Response: We appreciate the reviewer for raising this great point. Blocked Gibbs sampling indeed have been used with great success on SLDS. We would first like to point out the similarities of blocked Gibbs sampler and our closed-form distributional approach. In the current problems, blocked Gibbs sampler would cycle between sampling from $p(x_{1:T}^{(m)}|y_{1:T}, s_t = m\ \forall t, \theta^{(i-1)})$, and $p(s_t = m|y_{1:T}, x_{1:T}^{(m)}\ \forall m, \theta^{(i-1)})$, and from $p(\theta^{(i)}|y_{1:T}, s_t = m\ \forall t, x_{1:T}^{(m)}\ \forall m)$. As the reviewer suggested, weak, conjugate priors on the parameters would be made work very well. The blocked sampling of $p(x_{1:T}^{(m)}|y_{1:T}, s_t = m\ \forall t, \theta^{(i-1)})$ is done by forward-filtering-backward-sampling, as described in [Frühwirth-Schnatter, 1994, section 3.2]. Similarly, the probabilities of discrete states, $p(s_t = m|y_{1:T}, x_{1:T}^{(m)}\ \forall m, \theta^{(i-1)})$ can be found by forward-backward procedure similar to our work, and then a sample path can be generated [Carter and Kohn, 1994, Appendix 2]. Sampling from $p(\theta^{(i)}|y_{1:T}, s_t = m\ \forall t, x_{1:T}^{(m)}\ \forall m)$ is a bit trickier: variance parameters will have inverse gamma distribution, AR parameters will follow Gaussian distribution, oscillation frequency parameter will follow von Mises distribution and so on.

In comparison to that our generalized EM algorithm uses the same basic blocks, but instead of generating sample paths, it works directly with the actual probability distributions. For example, the E-step cycles between updating $p(x_{1:T}^{(m)}|y_{1:T}, s_t = m\ \forall t, \theta^{(i-1)})$ and $p(s_t = m|y_{1:T}, x_{1:T}^{(m)}\ \forall m, \theta^{(i-1)})$ in closed-form. $p(x_{1:T}^{(m)}|y_{1:T}, s_t = m\ \forall t, \theta^{(i-1)})$ is updated using forward-filtering-backward-smoothing algorithm

which only differs from forward-filtering-backward-sampling algorithm in the very last step: instead of generating sample path, it computes the mean and variance of the posterior distribution. Same remark follows for the $p(s_t = m|y_{1:T}, x_{1:T}^{(m)}\ \forall m, \theta^{(i-1)})$, with our algorithm stopping at the forward-backward procedure. Finally in the M-step the model parameters are updated using a set of closed-form update rules, instead of sampling from $p(\theta^{(i)}|y_{1:T}, s_t = m\ \forall t, x_{1:T}^{(m)}\ \forall m)$. So, there is no reason to believe that given the same initial parameters $\theta^{(0)}$, blocked Gibbs sampling would not result in similar estimates. In fact, when the posterior distributions of the states could not computed in closed-form (e.g., in case of a non-linear, Poisson observation model or when there is a dependency between $s_{t+1}^{(m)}$ and $x_t^{(m)}$), blocked Gibbs sampling would be crucial, greatly facilitated by resources such as PySLDS. We now point that out in the manuscript in **Discussion** (lines 526-529 p. 29).

We would also like to point out that 'the analog of interpolated density initialization' suggested by the reviewer is akin to initializing $g_t^{(m)}$ using the smoothed density, instead of using the interpolated density. That is because the samples from the conditional distribution $p(x_{1:T}^{(m)}|y_{1:T}, s_t = m\ \forall t, \theta^{(0)})$ will not reflect the deletion of y_t from $y_{1:T}$ when sampling x_t. We found the smoothed density to result in catastrophic failure of the fixed-point iterations to distinguish between candidate models, which in fact motivated us to derive the interpolated density. Our closed-form E-step gives the insight behind using interpolated density for the initialization, and makes it is easy to appreciate its role in the initialization. In a blocked Gibbs sampling setting, one can theoretically sample from the interpolated density derived in the paper, since the density parameters are composed of the quantities already computed in the forward-filtering-backward-sampling algorithm, and just run the very first blocked Gibbs sampling of $p(s_t = m|y_{1:T}, x_{1:T}^{(m)}\ \forall m, \theta^{(i-1)})$ considering the emission probabilities, $p(y_t \mid s_t = m, x_{1:T}^{(m)}\ \forall m, \theta^{(i-1)}) \propto \exp -|y_t - \check{y}_t^{(m)}|^2/2$, where $\check{y}_{1:T}^{(m)}$ is the block sample from the interpolated density. We omitted a direct comparison with sampling techniques since it is out of the scope of current work. Instead, we have added a discussion pointing readers to the possibility of using blocked Gibbs sampling for the inference in place of the Generalized EM algorithm in the **Discussion** section (lines 512-516, p. 29).

Concern 2: Framing of existing methods

The discussion presents existing works as "black-box variational inference" methods when in fact many of the cited references (39, 41, 71, 76) use structure exploiting inference algorithms like blocked Gibbs sampling (39, 41) or structured mean field approximations (71, 76). Both sets of algorithms exploit conditional conjugacy to update blocks of random variables at once, and they do so with exact updates rather than stochastic gradient ascent steps. The structured mean field approaches are directly analogous to the structured mean field approach used in this paper. The main difference is not black box vs structured, but rather the initialization scheme and the model/posterior family itself. T models and posterior approximation in this paper have a factorial construction with many continuous states evolving in parallel, rather than one continuous state with switching dynamics, as in the cited works.

Response: We would like to thank the reviewer for pointing out our misframing of the existing methods as all black-box variational inference. Indeed, much existing work exploits the structure of switching state-space models to derive efficient solutions. We have revised the discussion paragraphs (lines 506-548, pp. 28-30) to correctly present the main differences as in 1) model class, 2) initialization, and 3) sampling versus fixed-point iterations for state estimation. We would like to maintain the discussion on black-box variational inference per a previous suggestion from Reviewer 1, and we believe it is still a relevant inference approach; although as pointed out in ref 76, structured mean field approximation (e.g., variational Laplace EM) tends to perform better, which we highlight as a highly effective alternative to the solution we present here (lines 546-548, p. 30).

Concern 3: Switching SSM Framing in the Introduction

I disagree with the paragraph on lines 47-59 of the introduction.

– "These methods apply carefully constrained approximations to represent a broader range of switching processes besides the Markov process used in the HMM" My impression is that the cited work uses the same sorts of approximations as are developed in this submission, and they target very similar models (switching linear dynamical systems) with some extensions (nonparametric models, continuous-¿discrete dependencies, etc.)

- "Markov chain Monte Carlo sampling techniques are used to perform inference and learning for these models, which may be computationally burdensome for large data sets collected in neuroscience studies." The complexity of blocked Gibbs updates is identical to those of generalized EM. In practice, I've found blocked Gibbs often yields comparable estimates to structured mean field in the same wall-clock time, and can even be more robust to local optima than generalized EM.
- "Moreover, the nonparametric formulation of these algorithms may not be easily interpretable in relation to the neural mechanisms being studied." This seems quite speculative.
- "Similarly, under these methods it is difficult to constrain the switching inferences to candidate models formed based on clinical or neuroscience domain knowledge." I don't see why the cited models couldn't be constrained in the same way as done here.
- "Perhaps due to these challenges, these more recent Bayesian algorithms have also not been widely adopted to study neural data with time-varying dynamics." There has been a lot of recent work using switching linear dynamical systems to neural data with time-varying dynamics. In addition to the works cited already in the paper, see also:
  - Taghia et al (Nature Comm, 2019) https://www.nature.com/articles/s41467-018-04723-6
  - Nair et al (Cell, 2023) https://www.cell.com/cell/pdf/S0092-8674(22)01471-4.pdf
  - Large body of work from Emily Fox on Autoregressive HMMs for EEG analysis and Sandeep Datta's lab using AR-HMMs for behavioral modeling. These models are not the same as SLDS, but they are closely related and demonstrate the breadth with which switching state space models are used in neuroscience.

I realize this is just one paragraph, but I think it unfortunately misconstrues the existing work in this domain.

Response: We would like to again thank the reviewer for the careful read and point-by-point correction of this paragraph in the **Introduction**. We agree with the reviewer that the previous writing did not provide the correct framing of the more recent Bayesian methods and might have mislead readers away from using them, while our goal of this manuscript has been to advocate for broader and faster adoption of switching state-space models in neural signal processing. We have completely rewritten this paragraph and incorporated all points raised here by the reviewer (lines 47-70, pp. 4-5) along with the references pointed out (see [46-47] in pp. 56-57). We believe this has significantly strengthened our introduction of the existing literature and better contextualized our work.

# Concern 4: Balance of simulated vs real data examples

In my opinion, the most exciting part of this submission is not the algorithm derivation or the initialization scheme, but rather the application to spindle detection. I think it's very exciting to see these methods performing well on a non-trivial and clearly important scientific problem. Besides rebalancing the methodological section (Concern 1), I would personally prefer to see more emphasis placed on the applications and less on the relative comparison of initialization schemes.

Response: We appreciate the positive feedback on the spindle detection results. The **Results** sections are focused on comparing switching inference and learning performance with and without the interpolated density during initialization to highlight it as a key component of this work (pertaining to # Concern 1). We previously added the new simulation section on state-space oscillator models (p. 18) to highlight a new application example of switching state-space models following suggestions from Reviewer 1. This simulation study is meant to represent a realistic application scenario and emphasizes the potential of a proper algorithm to uncover subtle transitions between neural states. We feel strongly that the methodological background is necessary to motivate the importance of initialization and to provide complete derivations for applied users in the neuroscience field to more easily adopt the switching modeling framework. While an expanded analysis of exciting applications of switching state-space models in various EEG recordings is beyond the scope of the current work, we have modified our phrasing throughout the manuscript (e.g., lines 74-77, p. 5; line 101, p. 6, along with changes made to address # Concerns 1-3) in order to better frame our work as advocating for broader adoption of switching state-space models in

neural data analyses. An application of blocked Gibbs sampling and structured mean-field approximation to neural oscillations is mentioned as an exciting next step (lines 513-516, p. 29). In a future paper, we will focus on applying the method described here, along with blocked Gibbs sampling, block-box variational inference, and variational Laplace-EM in more concrete neuroscience problems. We would prefer to divert the emphasis on extracting neural dynamics and interpreting empirical results to this future work.

References

Christopher M. Bishop. *Pattern Recognition and Machine Learning.* Information Science and Statistics. Springer, New York, 2006. ISBN 978-0-387-31073-2.

Yaakov Bar-Shalom and Xiao-Rong Li. Estimation and tracking- Principles, techniques, and software. *Norwood, MA: Artech House, Inc, 1993.*, 1993.

Zoubin Ghahramani and Geoffrey E. Hinton. Variational Learning for Switching State-Space Models. *Neural Computation*, 12(4):831–864, April 2000. ISSN 0899-7667, 1530-888X. doi: 10.1162/089976600300015619.

Sylvia Frühwirth-Schnatter. DATA AUGMENTATION AND DYNAMIC LINEAR MODELS. *Journal of Time Series Analysis*, 15(2):183–202, March 1994. ISSN 0143-9782, 1467-9892. doi: 10.1111/j.1467-9892.1994.tb00184.x.

C. K. Carter and R. Kohn. On Gibbs sampling for state space models. *Biometrika*, 81(3):541–553, 1994. ISSN 0006-3444, 1464-3510. doi: 10.1093/biomet/81.3.541.
